# Effect of environmental factors on seminal microbiome and impact on sperm quality

**DOI:** 10.3389/fendo.2024.1348186

**Published:** 2024-02-22

**Authors:** Filipe T. Lira Neto, Marina C. Viana, Federica Cariati, Alessandro Conforti, Carlo Alviggi, Sandro C. Esteves

**Affiliations:** ^1^ AndrosRecife, Andrology Clinic, Recife, Brazil; ^2^ ANDROFERT, Andrology and Human Reproduction Clinic, Campinas, Brazil; ^3^ Department of Public Health, University of Naples Federico II, Napoli, Italy; ^4^ Department of Neuroscience, Reproductive Science and Odontostomatology, University of Naples, Federico II, Naples, Italy; ^5^ Department of Surgery (Division of Urology), University of Campinas (UNICAMP), Campinas, Brazil; ^6^ Department of Clinical Medicine, Faculty of Health, Aarhus University, Aarhus, Denmark

**Keywords:** male infertility, microbiome, microbiota composition, semen, spermatozoa, testicular microbiome, testis

## Abstract

**Objective:**

This review provides a comprehensive overview of the existing research on the seminal microbiome and its association with male infertility, while also highlighting areas that warrant further investigation.

**Methods:**

A narrative review was conducted, encompassing all relevant studies published between 1980-2023 on the male reproductive tract microbiome in humans. This review considered studies utilizing culture-based, polymerase chain reaction (PCR)-based, and next-generation sequencing (NGS)-based methodologies to analyze the microbiome. Data extraction encompassed sample types (semen or testicular tissue), study designs, participant characteristics, employed techniques, and critical findings.

**Results:**

We included 37 studies comprising 9,310 participants. Among these, 16 studies used culture-based methods, 16 utilized NGS, and five employed a combination of methods for microorganism identification. Notably, none of the studies assessed fungi or viruses. All NGS-based studies identified the presence of bacteria in all semen samples. Two notable characteristics of the seminal microbiome were observed: substantial variability in species composition among individuals and the formation of microbial communities with a dominant species. Studies examining the testicular microbiome revealed that the testicular compartment is not sterile. Interestingly, sexually active couples shared 56% of predominant genera, and among couples with positive cultures in both partners, 61% of them shared at least one genital pathogen. In couples with infertility of known causes, there was an overlap in bacterial composition between the seminal and vaginal microbiomes, featuring an increased prevalence of Staphylococcus and Streptococcus genera. Furthermore, the seminal microbiome had discernible effects on reproductive outcomes. However, bacteria in IVF culture media did not seem to impact pregnancy rates.

**Conclusion:**

Existing literature underscores that various genera of bacteria colonize the male reproductive tract. These organisms do not exist independently; instead, they play a pivotal role in regulating functions and maintaining hemostasis. Future research should prioritize longitudinal and prospective studies and investigations into the influence of infertility causes and commonly prescribed medication to enhance our understanding of the seminal microbiota’s role in reproductive health.

## Introduction

Contrary to earlier perceptions that primarily portrayed bacteria as pathogenic adversaries, contemporary insights reveal a fascinating truth: the human body is teeming with more bacteria than human cells (1). This revelation aligns with the recognition that nearly all organs and systems host a companion microbiota composed of bacteria, fungi, and viruses that coexist harmoniously with human hosts (2). These organisms do not lead solitary lives; instead, they play a pivotal role in regulating bodily functions and maintaining hemostasis. Perturbations in the microbiota, termed dysbiosis, which can encompass imbalances in microbial community composition, loss of beneficial symbionts, proliferation of pathobionts or opportunistic organisms, and disruptions in inter-microbial competition and diversity, have been implicated in the onset or exacerbation of various diseases (3).

The term ‘microbiome’ refers to diverse microorganisms inhabiting specific organs, systems, or biofluids. Next-generation sequencing (NGS) technology has ushered in a new era of understanding the human microbiome, enabling the detection of previously unknown commensal and pathogenic microorganisms (4). Leveraging this high-throughput technique, the ‘Human Microbiome Project’ has characterized microbiomes in various bodily organs and has reported that the urogenital tract microbiome constitutes approximately 9% of the total human microbiota (5, 6). Notably, dysbiosis of the female reproductive microbiome has been associated with reduced pregnancy rates and adverse pregnancy outcomes (7).

Despite considerable progress in elucidating the human microbiome, the characterization of the male genital tract microbiome remains in its early stages. Most studies concerning the male reproductive microbiota center on the seminal microbiome. Semen comprises secretions from the testicles, epididymis, prostate, seminal vesicles, bulbourethral glands, and periurethral glands, providing a conducive environment for microbial growth due to its nutrient content (8, 9). Therefore, the seminal microbiome serves as a representative of the entire male genital system.

A male factor is identified in up to 50% of infertile couples, and urogenital tract infection represents a potential etiological contributor (10, 11). Additionally, as many as 25% of men with abnormal semen analysis results are categorized as having idiopathic infertility due to the absence of discernible causes using current diagnostic tools (7, 12). Beyond the conventional mechanisms by which pathogenic bacteria can adversely affect male fertility, such as impairing sperm motility and capacitation and inducing oxidative stress and apoptosis (13–21), some researchers propose that dysbiosis of the seminal microbiome may also exert adverse effects on male fertility through as-yet-unclear pathways (2).

To consolidate the evidence concerning the seminal microbiome and its association with male infertility, we conducted a comprehensive narrative review of all studies about the male reproductive tract microbiome in humans from 1980 to 2023. Our review encompassed research that employed various methodologies, including culture-based, polymerase chain reaction (PCR)-based, and NGS-based techniques to investigate the microbiome in human semen or testicular tissue samples. We systematically collected information on sample types (semen or testicular tissue), study designs, participant demographics, employed methodologies, and key findings. To assess the quality of the included studies, we utilized the ‘Study Quality Assessment Tool for Before-After (Pre-Post) Studies with no Control Group’ developed by the National Heart, Lung, and Blood Institute. The eligible studies were categorized into three tiers based on their quality: high, medium, or low (www.nhlbi.nih.gov/health-topics/study-quality-assessment-tools). Our review also explores the influence of environmental factors on the seminal microbiome. Finally, we examine the evolving clinical practices stemming from this emerging knowledge and recommend topics for future research to address the existing knowledge gaps.

## How to assess the microbiome

The initial investigations into the bacterial content of semen relied on culture-based techniques primarily targeting well-known pathogenic bacteria, like *Staphylococcus, Enterococcus, Escherichia, and Ureaplasma* (22–24). Consequently, these earlier endeavors yielded limited insights into the resident seminal microbiota, particularly concerning anaerobes and fastidious bacteria, which are challenging to cultivate (5). Subsequently, PCR-based studies made strides in identifying a broader spectrum of bacteria genera. However, they still failed to provide a comprehensive overview of the seminal microbiome (25). This limitation arose from the requirement to predetermine the genera of bacteria under investigation, rendering the technique less effective for polymicrobial specimens and frequently resulting in data that were challenging to interpret (26).

The emergence of NGS technology marked a remarkable breakthrough in exploring the human microbiome. This method directly sequences microbial DNA or RNA within samples, eliminating the reliance on traditional culture-based approaches (4). Two primary NGS techniques employed for microbiome characterization are amplicon sequencing and shotgun metagenomic sequencing (27).

Amplicon sequencing involves amplifying a specific region of DNA through PCR and then sequencing the resultant product. Typically, this entails targeting one or more hypervariable regions of the bacterial 16S ribosomal RNA (rRNA) gene (4). The hypervariable regions, being highly conserved and ubiquitous among bacteria, offer a suitable basis for analysis (28). Nevertheless, due to practical constraints related to time and cost, only a subset of these variable regions is generally chosen for sequencing. This approach introduces potential bias since no single region effectively distinguishes all bacteria species, and sequencing specific hypervariable regions may yield varying results.

In contrast, shotgun metagenomic sequencing (SMS) comprehensively assesses all the DNA within a given sample. This method involves DNA extraction and random fragmentation, followed by the ligation of barcodes and adapters to each fragment, facilitating sample identification and DNA sequencing. Subsequently, the obtaining reads are meticulously cleaned and aligned with a reference database to identify taxa and assess functional potential (28). Unlike amplicon sequencing, SMS metagenomic sequencing enables the detection of fungi, parasites, and DNA viruses (29). Furthermore, SMS has superior resolution and sensitivity in detecting species-level changes and predicting functional potential (28).

## Seminal microflora of healthy men

A limited number of studies employing NGS have investigated the seminal microbiome of healthy men, often including them as part of a control group (**Table 1**). Notably, two distinct features have emerged regarding the seminal microbiome: a wide variation in species composition among individuals and the formation of microbial communities dominated by particular species (2, 22).

**Table 1:** Characteristics of studies examining the seminal microbiome in healthy men.

Author, year, (country)	Design	Patients	Sample type	Technique	Main Phyla/Genera	Other findings	Study quality
Veneruso et al., 2023 (30) (Italy)	Cross-sectional	7 men with normal SA	Semen	Sequencing V4 – V6 hypervariable region of 16S rRNA gene	Proteobacteria were the most abundant phylum; *Achromobacte, Staphylococcus, Gardnerella*, and *Serratia* were the most abundant genera		High
Yao et al., 2022 (31) (China)	Cross-sectional	20 men with normal SA	Semen	Sequencing V3 - V4 hypervariable region of 16S rRNA gene	*Streptococcus, Lactobacillus, Burkholderia-Caballeronia-Paraburkholderia, Staphylococcus, Gardnerella* *Lactobacillus*-enriched group predominated in men with normal SA *Streptococcus*-enriched group predominated in men with leukocytospermia		High
Bukharin et al., 2022 (32) (Russia)	Cross-sectional	30 healthy men	Semen	Culture + Sequencing of 16S rRNA gene)	*Staphylococcus, Corynebacterium, Enterococcus, Neisseria, Veillonella;*		Fair
Lundy et al., 2021 (33) (USA)	Cross-sectional	12 men with proven paternity	Semen Urine Rectal swab	Sequencing V3 - V4 hypervariable region of 16S rRNA gene)	Urine and semen contained an abundance of *Gardnerella* and *Corynebacterium* compared to the rectum Decreased *Veillonella, Prevotella* and increased *Pseudomonas, Pseudoxanthomonas*, and *Acidovorax* in semen compared to urine	*Collinsella* and *Staphylococcus* depleted in semen following vasectomy; *Finegoldia* in uncircumcised men	High
Pagliuca et al., 2021 (16) (Italy)	Cross-sectional	16 men with normal SA	Semen	Culture positive if concentration > 10³cfu/m and PCR	*Staphylococcus coagulase negative, Enterococcus faecalis, Streptococcus anginosus, Streptococcus agalactiae, Staphylococcus aureus*		High
Okwelogu et al., 2021 (34) (Nigeria)	Cohort	11 men with normal basic SA	Semen	Sequencing V4 hypervariable region of 16S rRNA gene)	*Lactobacillus, Gardnerella, Veillonella, Corynebacterium, Escherichia, Haemophilus, Prevotella*	Couples shared 56% of the predominant genera; Couples with clinical pregnancy after IVF had increased abundance of *Lactobacillus jensenii, and Faecalibacterium*, whereas *Proteobacteria, Prevotella,and Bacteroidetes were decreased*	High
Yang et al., 2020 (35) (China)	Cross-sectional	58 healthy controls	Semen	Sequencing V1 and V2 hypervariable region of 16S rRNA gene)	*Pelomonas, Propionibacterium, Boseagenosp, Bosea, Afipia, Sphingomonas*, and *Vogesella*		Fair
Baud et al., 2019 (36) (Switzerland)	Cross-sectional	26 men with normal SA	Semen	Sequencing V1 and V2 hypervariable regions of 16S rRNA gene)	Overall: *Actinobacteria, Bacteroidetes, Firmicute*, and *Proteobacteria phyla;* *Staphylococcus* genus was significantly more abundant in the normal SA group*;* *Lactobacillus* genus was enriched in samples with normal morphology	Three broad microbiota profiles identified: *Prevotella*-dominant, *Lactobacillus*-dominant, and Polymicrobial	High
Alfano et al., 2018 (37) (Italy)	Cross-sectional	5 men with normal spermatogenesis who underwent orchiectomy	Testicular tissue	Sequencing V3 to V5 hypervariable regions of 16S rRNA gene)	Normal germline: *Actinobacteria, Bacteroidetes, Firmicutes*, and *Proteobacteria*		High
Zeyad et al., 2018 (38) (Germany)	Cross-sectional	55 non-bacteriospermic men	Semen	Culture: positive if concentration > 10³cfu/ml	*S. aureus (9%), E. coli (7%)*, *S epidermidis (6%)*, *S haemolyticus (5%)*, *E. faecalis (5%)*, and *S. agalactiae (2%)*	No significant DFI differences in men with bacteriospermia; Decreased fertilization in men with bacteriospermia (p<0.05)	High
Chen et al., 2018 (39) (China)	Cross-sectional	5 fertile semen donors	Semen	Sequencing V4 hypervariable regions of 16S rRNA gene	Firmicutes, Proteobacteria, Bacteroidetes and Actinobacteria were the predominant phyla *Lactobacillus, Prevotella, Proteus, Pseudomonas*, and *Veillonella* were the dominant genera	*Alicyclobacillus, Amaricoccus, Anaeromyxobacter, Aquicella, Arsenicicoccus* were decreased when compared to men with azoospermia	High
Monteiro et al., 2017 (40) (Portugal)	Cross-sectional	29 men with normal basic SA	Semen (pooled by subgroups)	Sequencing V3 to V6 hypervariable regions of 16S rRNA gene)	Overall: *Enterococcus, Staphylococcus, Anaerococcus, Corynebacterium, Peptoniphilus*, and *Propionibacterium*	*Corynebacterium*, *Haemophilus*, and *Streptococcus* at considerable abundancies (>1%)	High
Vilvanathan et al., 2016 (41) (India)	Cross-sectional	47 men with normal sperm count	Semen	Culture: positive if concentration > 10³cfu/ml	Overall: *E. faecalis (30%)*, *Coagulase negative Staphylococcus (23.3%)*, *Staphylococcus aureus (20%)*, *E. coli (10%)*, *Klebsiella pneumoniae (6.6%)*, *Proteus sp (6.6%)*, and *Citrobacter sp (3.3%)*		High
Fraczek et al., 2016 (42) (Poland)	Cross-sectional	30 normozoospermic men without bacteriospermia or leucocytospermia	Semen	Culture: positive if concentration > 10⁴cfu/ml	Coagulase-negative *Staphylococcus (22.9%), Streptococcus spp (12.3%), Enterococcus spp (13.8%), Mycoplasma spp (4.6%);* Gram+ aerobic (16.5%): *Corynebacterium glucuronolyticum-seminale, C. striatum*, and *C. propinquum* Gram-negative aerobic (3.7%): *Escherichia coli*, and *Proteus mirabilis)* Gram+ anaerobic (6.4%): *Propionibacterium acnes, P. propionicum, P. avidum*, and *Bifidobacterium* sp. Gram negative anaerobic (13.8%): *Bacteroides urealyticum, Prevotella melaninogenica, P. intermedia*, and *Fusobacterium varium;* One sample*: Candida albicans*		High
Weng et al., 2014 (27) (China)	Cross-sectional	10 men with abnormal semen volume	Semen	Sequencing V4 hypervariable region of 16S rRNA gene	Overall: *Lactobacillus, Pseudomonas, Prevotella, Gardnerella, Rhodanobacter, Streptococcus, Finegoldia* and *Haemophilus* Normal SA group: *Lactobacillus, Pseudomonas, Gardnerella, Prevotella Rhodanobacter*, and *Streptococcus*,		High
Hou et al., 2013 (43) (China)	Cross-sectional	19 healthy sperm donors	Semen	Sequencing V1 and V2 hypervariable regions of 16S rRNA gene	Overall: *Ralstonia, Lactobacillus, Corynebacterium, Streptococcus, Staphylococcus, Prevotella, Finegoldia*, and *Anaerococcus*		Fair
Kjaergaard et al., 1997 (44) (Denmark)	Cross-sectional	115 normozoospermic men	Semen	Culture positive if concentration >10³cfu/m and PCR	Commensals: *Ureaplasma urealyticum*, *Gardnerella vaginalis*, *Enterococcus faecalis*, and Enterobacteriaceae	No association between semen quality and semen microorganisms	High

SA, semen analysis; DFI, Sperm DNA Fragmentation Index.

In one of the initial NGS-based studies, Hou et al. sequenced the V1-V2 regions of 16S rRNA genes, revealing that even in healthy sperm donors, semen harbors a more diverse bacteria population than sperm itself (43). Their findings highlighted *Ralstonia, Lactobacillus, Corynebacterium, Streptococcus, and Staphylococcus* as the most prevalent bacteria in seminal fluid; these bacteria are organized into six distinct communities based on species composition and structure.

Weng et al. employed sequencing of the V4 hypervariable region of the 16S rRNA gene to examine 36 semen samples with normal basic semen analysis parameters. Their study identified *Lactobacillus, Pseudomonas, Gardnerella, Prevotella, and Rhodanobacter* as the most common genera (27). Additionally, the bacterial communities formed three main clusters: *Pseudomonas*-predominant, *Lactobacillus*-predominant, and *Prevotella*-predominant, with *Lactobacillus*-predominant group being the most frequent in the normal samples.

Similarly, Baud et al., utilizing sequencing of the V1 and V2 hypervariable regions of the 16S rRNA gene, examined 26 samples from men undergoing fertility evaluation who had normal basic semen analysis parameters. Their findings revealed three distinct microbiota communities: a *Lactobacillus*-predominant group, a *Prevotella*-predominant group, and a polymicrobial group (36). *Staphylococcus* was associated with normal semen analysis parameters, while the *Lactobacillus* genus was enriched in samples with normal morphology.

Another study reported that *Lactobacillus*, *Gardnerella*, *Veillonella*, *Corynebacterium*, and *Escherichia* were the most prevalent genera in the semen of men with normal basic semen analysis results (34). Bukharin et al. analyzed the seminal microbiome composition in 30 healthy men, identifying *Staphylococcus*, *Corynebacterium*, *Enterococcus*, *Neisseria*, and *Veillonella* as the most prevalent genera (32). Moreover, Yao et al. examined semen samples of 20 men with normal basic semen analysis parameters, revealing the main genera as *Streptococcus*, *Lactobacillus, Burkholderia-Caballeronia-Paraburkholderia*, *Staphylococcus* and *Gardnerella* (31). Interestingly, a *Lactobacillus*-enriched group predominated among these men.

Conversely, Monteiro et al. reported a low prevalence of *Lactobacillus* and a high prevalence of *Enterococcus* in semen samples of men with normal basic semen analysis parameters, employing sequencing of the V3 to V6 hypervariable regions of the 16S rRNA gene (40). However, it is essential to note that all the samples used in this study were derived from leftovers of assisted reproduction procedures, making it possible that some cases might have involved male factor infertility. Correspondingly, Yang et al., using sequencing of the V1 and V2 hypervariable regions of the 16S rRNA gene, demonstrated that *Pseudomonas*, *Propionibacterium*, *Boseagenosp*, *Bosea*, and *Afipia* were the most prevalent genera in healthy men with normal basic semen analysis parameters (35). Intriguingly, these authors observed an increased abundance of *Lactobacillus* in men with abnormal semen analysis results.

**Figure 1:**
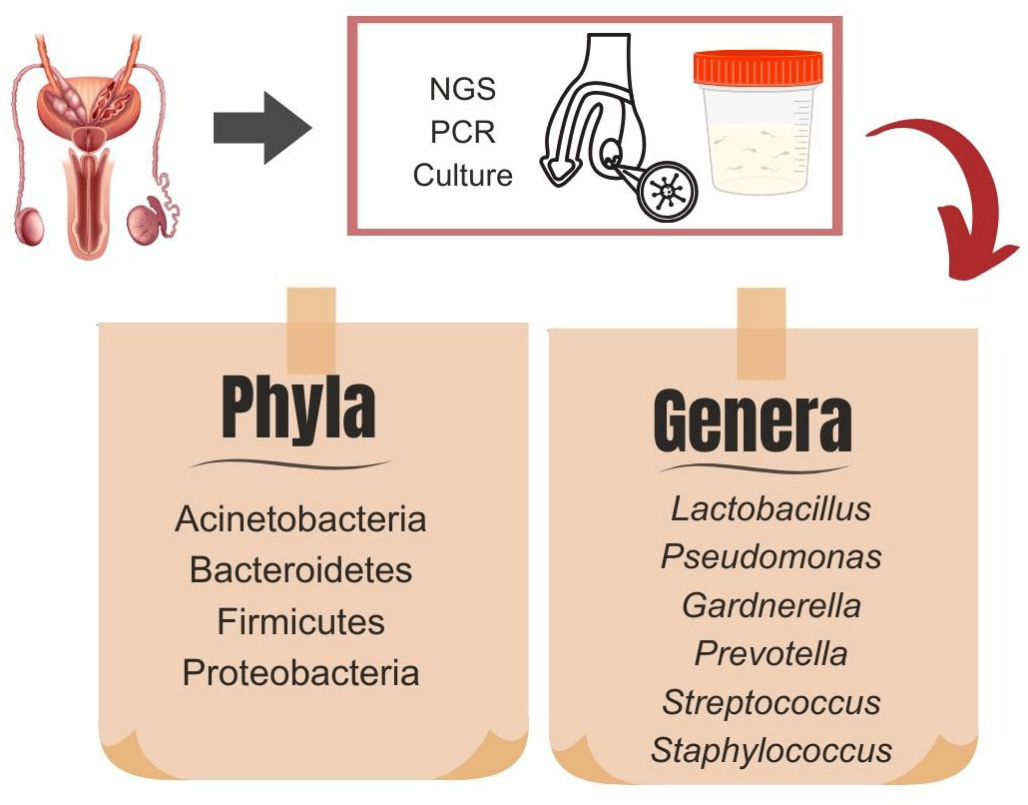
Dominant microbiota phyla and genera in testicular tissue samples and semen analysis obtained by existing diagnostic methods: next-generation sequencing (NGS), culture, and polymerase chain reaction (PCR).

Given the diverse microfluidic components of semen and the microbiome’s complexity, it is estimated that approximately 30% of microorganisms in the semen originate from the urethra microbiome (35). Furthermore, specific genera, such as *Pseudomonas*, *Pseudoxanthomonas*, and *Acidovorax*, are overrepresented in the seminal microbiome compared to the urethral microbiome, suggesting their origin from upstream anatomic compartments (33). Thus, the seminal microbiome represents a composite of the microbiomes of the testicular, epididymal, prostatic, vesicular, and urethral regions (33) (**Figure 1**). Higher microbiota diversity in the gut, skin, and oral cavity is often considered beneficial for human health (45). Interestingly, data from studies regarding the male genital tract microbiome is heterogeneous. Some authors have suggested that greater microbiota diversity harms sperm health (32, 46, 47), while others have found that reduced seminal biodiversity is associated with poor semen quality (30, 39).

## Seminal microflora of men with altered semen quality

**Figure 2:**
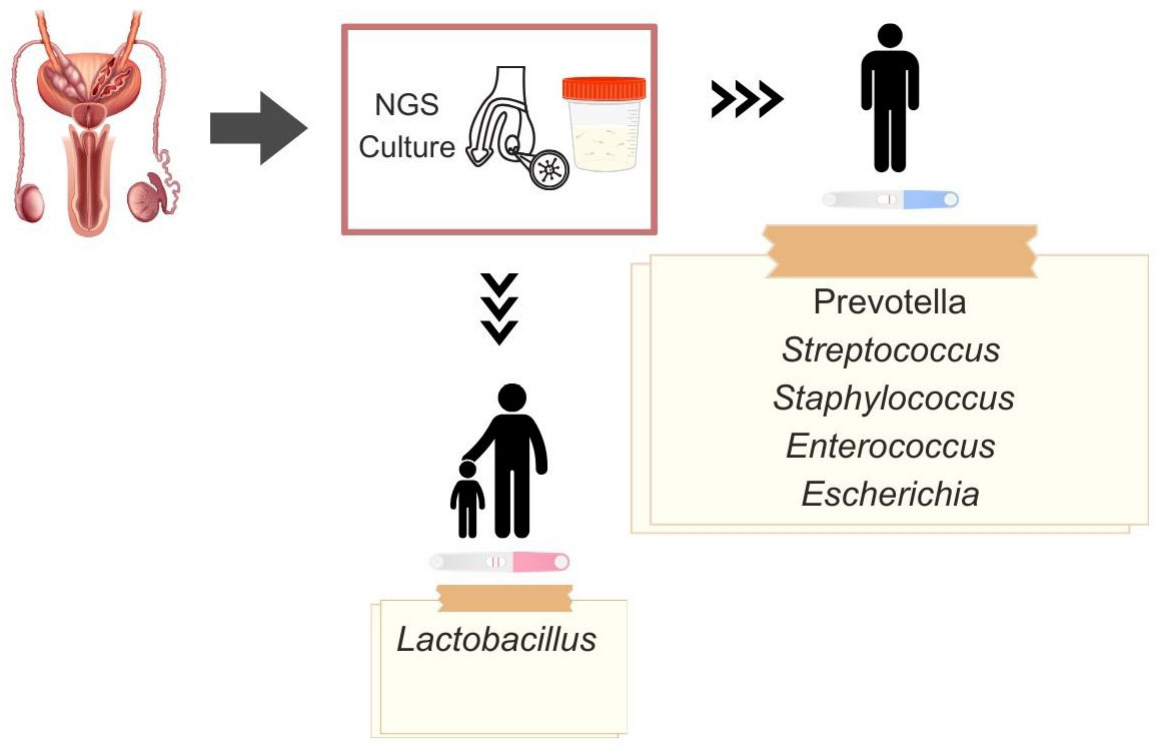
Dominant microbiota genera in fertile and infertile men, obtained by existing diagnostic methods: next-generation sequencing (NGS), culture, and polymerase chain reaction (PCR).

Most culture-based studies examining cohorts of infertile couples have failed to establish a conclusive link between the presence of bacteria in semen and abnormal semen analysis parameters (33, 41, 48–51). However, Ricci et al. reported reduced sperm motility in samples testing positive for microorganisms compared to negative samples (52). They also observed a negative association between *E. faecalis* and semen quality. Likewise, Zeyad et al. identified a negative impact of bacterial presence on sperm concentration and motility (38). Along these lines, Pagliuca et al. showed a significant correlation between infected status assessed by culture and PCR with semen volume, sperm concentration, and motility (16). Below, we summarize findings from studies using NGS to explore the microbiome of men with abnormal semen analysis parameters (**Figure 2** **;** **Table 2**).

**Table 2:** Characteristics of studies investigating semen/sperm microbiome in infertile men.

Author, year, (country)	Design	Patients	Sample type	Technique	Main Phyla/Genera	Other findings	Study quality
Veneruso et al., 2023 (30) (Italy)	Cross-sectional	13 men with abnormal SA	Semen	Sequencing V4 – V6 hypervariable region of 16S rRNA gene	Proteobacteria were the most abundant phylum; *Lactobacillus, Escherichia, Shigella*, and *Serratia* were the most abundant genera	The genera *Mannheimia, Escherichia_Shigella*, and *Varibaculum* were significantly increased in men with abnormal SA when compared to men with normal SA	High
Yao et al., 2022 (31) (China)	Cross-sectional	13 men with asthenozoospermia, 22 men with leukocytospermia, and 32 men with asthenozoospermia and leukocytospermia;	Semen	Sequencing V3 - V4 hypervariable region of 16S rRNA gene	Overall: *Streptococcus, Lactobacillus, Burkholderia-Caballeronia-Paraburkholderia, Staphylococcus*, and *Gardnerella;* *Lactobacillus*-enriched group predominated in men with asthenozoospermia, whereas *Streptococcus*-enriched group predominated in men with leukocytospermia	Diversity increased in men with leukocytospermia; Bacteroides were increased in men with leukocytospermia	High
Bukharin et al., 2022 (32) (Russia)	Cross-sectional	42 infertile men with abnormal SA	Semen	Culture + Sequencing of 16S rRNA gene	*Staphylococcus, Corynebacterium, Enterococcus, Streptococcus*, and *Escherichia*		Fair
Molina et al., 2021 (53) (Spain)	Cross-sectional	7 azoospermic men (13 samples), 3 men with high SDF (9 samples), and 1 man with severe OAT (2 samples)	Testicular tissue	Sequencing V3 and V4 hypervariable regions of 16S rRNA gene	*Blautia, Cellulosibacter, Clostridium XIVa, Clostridium XIVb, Clostridium XVIII, Collinsella, Prevotella, Prolixibacter, Robinsoniella*, and *Wandonia.*	50-70% contamination	High
Lundy et al., 2021 (33) (USA)	Cross-sectional	25 men with primary idiopathic infertility	Semen, Urine, and Rectal swab	Sequencing V3 - V4 hypervariable region of 16S rRNA gene	Infertile group: Increased *Aerococcus, Prevotella, Pseudomonas*, and decreased *Collinsella* Infertile group + varicocele*: Bacteroids, Peptoniphilus*	Rectum of infertile men: decreased A*naerococcus* and increased *Lachnospiraceae, Collinsella*, and *Coprococcus;* Urine of infertile men: increased *Anaerococcus;* SAM cycle strongly over-represented in the urine and semen of infertile men	High
Pagliuca et al., 2021 (16) (Italy)	Cross-sectional	37 men with abnormal SA	Semen	Culture positive if concentration > 10³cfu/m and PCR	*Staphylococcus coagulase negative, Haemophilus haemolyticus, Enterococcus faecalis, Haemophilus parainfluenzae, Gardnerella vaginalis*	Bacteria were found more frequently in men with abnormal SA when compared to those with normal SA (70% vs 31%)	High
Okwelogu et al., 2021 (34) (Nigeria)	Cohort	36 male partners of infertile couples: 7 men with oligozoospermia, 7 men with azoospermia, 10 men with asthenozoospermia, and 1 man with teratozoospermia	Semen	Sequencing V4 hypervariable region of 16S rRNA gene	Oligozoospermia: *Prevotella, Escherichia, Lactobacillus, Shuttleworthia, Serratia, Megasphaera, Gardnerella, Sneathia, Porphyromonas;* Azoospermia: *Lactobacillus, Enterococcus, Corynebacterium, Veillonella, Gardnerella, Ureaplasma*, and *Prevotella*	Leukocytospermia: Increased *Bacteroides* and *Prevotella;* Decreased *Lactobacillus reuteri* group, and *Faecalibacterium*	High
Campisciano et al., 2020 (54) (Italy)	Cohort	47 male partners of infertile couples: 22 men with explained infertility, and 25 with unexplained infertility	Semen	Sequencing V3 hypervariable region of 16S rRNA gene	Overall: *Prevotella* Explained Infertility group: Increased Prevotella (*p. bivia and Staphylococcus*); Unexplained Infertility group: Increased *Lactobacillus gasseri*	*Prevotella* had a higher relative abundance in HPV-positive semen samples (25% vs. 17%)	High
Yang et al., 2020 (35) (China)	Cross-sectional	8 men with azoospermia, 58 men with asthenozoospermia, and 22 men with oligoasthenozoospermia	Semen	Sequencing V1 and V2 hypervariable region of 16S rRNA gene	Men with asthenozoospermia had increased abundance of *Sneathia, Ralstonia, Ureaplasma*, *Bacteroides*, and *Chryseobacterium* Men with oligoasthenozoospermia had an increased abundance of *Ralstonia, Oscillospira, Parabacteroides, Lachnospira*, and *Phascolarctobacterium*		Fair
Baud et al., 2019 (36) (Switzerland)	Cross-sectional	68 men with abnormal SA	Semen	Sequencing V1 and V2 hypervariable regions of 16S rRNA gene	*Prevotella* genus was significantly enriched in the abnormal SA group	Three broad microbiota profiles identified: *Prevotella*-dominant, *Lactobacillus*-dominant, and Polymicrobial	High
Ndiokwere et al., 2019 (55) (Nigeria)	Cross-sectional	22 semen samples from men undergoing fertility evaluation	Semen	Sequencing V4 hypervariable region of 16S rRNA gene	*Serratia, Lactobacillus, Corynebacterium, Staphylococcus*, and *Prevotella*	Species*: Serratia marcescens Lactobacillus iners, Serratia entomophila, Haemophilus parainfluenzae*, and *Corynebacterium tuberculostearicum*	Fair
Zeyad et al., 2018 (38) (Germany)	Cross-sectional	29 men with bacteriospermia	Semen	Culture: positive if concentration > 10³cfu/ml	*S. aureus* (9%), *E. coli* (7%), *S. epidermidis* (6%), *S. haemolyticus (*5%), *E. faecalis* (5%), and *S. agalactiae (2%)*	Bacteriospermia 34.5% of samples; Bacteriospermia associated with reduced sperm concentration and motility; Bacteriospermia not associated with increased DFI; Bacteriospermia associated with decreased fertilization	High
Ricci et al., 2018 (52) (Italy)	Cross-sectional	285 male partners of infertile couples	Semen	Culture positive if concentration > 10³cfu/m	Bacteriospermia in 29.1% of specimens; *Staphylococcus aureus* (0.7%), *Enterococcus fecalis* (11.6%), *Streptococcus agalactiae* (4.6%), *Escherichia coli* (6.7%), *Streptococcus anginosus* (0.3%), *S. haemolyticus* (2%), *and U. urealyiticum* (2%)	Bacteriospermia associated with a decrease in total motility and progressive motility; *Enterococcus fecalis* associated with reduced sperm motility and morphology	High
Chen et al., 2018 (39) (China)	Cross	6 men with OA; 6 men with iNOA	Semen	Sequencing V4 hypervariable regions of 16S rRNA gene	Firmicutes, Proteobacteria, Bacteroidetes and Actinobacteria were the predominant phyla *Lactobacillus, Prevotella, Proteus, Pseudomonas*, and *Veillonella* were the dominant genera	*Solibacillus, Campylobacter, Campyiobacteraceae and Plesiomonas* were reduced in the OA group; *Sneathia* and *Lysobacter* were reduced in iNOA group	High
Alfano et al., 2018 (37) (Italy)	Cross-sectional	10 men with iNOA: 5 with positive sperm retrieval, and 5 with negative sperm retrieval	Testicular tissue	Sequencing V3 to V5 hypervariable regions of 16S rRNA gene	*Actinobacteria* and *Firmicutes*	Increased number of bacteria in the testis of iNOA men; Positive sperm retrievals: *Actinobacteria* and *Firmicutes* Negative sperm retrievals: *Actinobacteria*	High
Zeyad et al., 2017 (56) (Germany)	Cross-sectional	36 men with bacteriospermia	Semen	Culture: positive if concentration > 10³cfu/ml	*Staphylococcus sp* (15%; *aureus, epidermidis, haemolyticus, xylosus)*; *Escherichia coli* (5%); *Streptococcus spp* (6%: *agalactie, pneumoniae);* *Enterococcus faecalis* (4%), and *Klebsiella pneumoniae* (1.6%)	Bacteriospermia associated with reduced sperm concentration and motility; Neither morphology nor DFI was significantly impacted by bacteriospermia	High
Monteiro et al., 2017 (40) (Portugal)	Cross-sectional	27 men with AT, 35 men with OAT, And 27 men with hyperviscosity	Semen (pooled by subgroups)	Sequencing V3 to V6 hypervariable regions of 16S rRNA gene	Overall: *Enterococcus, Staphylococcus, Anaerococcus, Corynebacterium, Peptoniphilus*, and *Propionibacterium;* OAT and Hyperviscosity groups: *Cyanobacteria* and *Fusobacteria*	Lower prevalence of *Lactobacillus* and *Propionibacterium*; Higher prevalence of *Pseudomonas*, *Klebsiella*, *Aerococcus*, *Actinobaculum*, and *Neisseria* in OAT and hyperviscosity groups	High
Vilvanathan et al., 2016 (41) (India)	Cross-sectional	37 men with oligozoospermia and 1 individual with azoospermia	Semen	Culture: positive if concentration > 10³cfu/ml	Bacteriospermia in 35% of specimens; Overall: *E. faecalis* (30%), *Coagulase-negative Staphylococcus* (23.3%), *Staphylococcus aureus* (20%), *E. coli* (10%), *Klebsiella pneumoniae* (6.6%), *Proteus sp* (6.6%), and *Citrobacter sp* (3.3%)	Presence of asymptomatic bacteriospermia not associated with abnormal semen parameters; Altered semen quality among different bacterial species lacked significant associations	Fair
Mashaly et al., 2016 (57) (Egypt)	Cross-sectional	60 infertile men: 30 without leukocytospermia (G1), and 30 with leukocytospermia (G2)	Semen	Culture: positive if concentration > 10.000 cfu/ml	G1: *Corynebacterium* (26.7%), *Corynebacterium + E. coli* (3.3%), *Staphylococcus aureus* (13.3%), *Haemolytic streptococci + E.coli* (3.3%); G2: *Corynebacterium* (10%), *Corynebacterium + E.coli* (0%), *Staphylococcus aureus* (10%), *Haemolytic streptococci + E.coli* (0%)	Bacteriospermia in 33% of specimens; 20% *Corynebacteria;* Sperm motility considerably lower in positive culture with *Corynebacteria*; Nonsignificant difference in sperm concentration and morphology between patients with Corynebacteria positive or negative cultures	High
Ruggeri et al., 2016 (58) (Italy)	Cross-sectional	246 male partners of infertile couples: 212 negative semen culture; 15 positive semen culture; 19 mixed flora	Semen	Not specified	*Enterococcus faecalis* most common in both men (2.8%) and women (3.6%); *Escherichia coli:* men (0.8%) vs. women (3.2%); *Ureaplasma urealyticum:* 3.2% (men)		High
Fraczek et al., 2016 (42) (Poland)	Cross-sectional	30 normozoospermic men with isolated bacteriospermia; 22 normozoospermic with bacteriospermia and leukocytospermia; 19 normozoospermic with isolated leukocytospermia;	Semen	Culture: positive if concentration > 10⁴cfu/ml	Coagulase-negative: *Staphylococcus* (22.9%), *Streptococcus spp* (12.3%), *Enterococcus spp* (13.8%), *Mycoplasma spp* (4.6%), *Gram+ aerobic* (16.5%)*, Corynebacterium glucuronolyticum-seminale, C. striatum*, and *C. propinquum;* Gram negative aerobic (3.7%): *Escherichia coli*, and *Proteus mirabilis);* Gram+ anaeroibic (6.4%): *Propionibacterium acnes, P. propionicum, P. avidum, Bifidobacterium* sp.*)* Gram negative anaerobic (13.8%): *Bacteroides ureolyticum, Prevotella melaninogenica, P. intermedia*, and *Fusobacterium varium* One sample*: Candida albicans*	Reduced sperm concentration in all groups compared to the control group; Significant sdeterioration of motility in the isolated leucocytospermia group; Necrozoospermia significantly higher in the combined bacteriospermia + leucocytospermia group; Teratozoospermia significantly higher in the isolated bacteriospermia group	Fair
Mändar et al., 2015 (59) (Estonia)	Cross-sectional	23 infertile men	Semen	Sequencing V6 hypervariable region of 16S rRNA gene	*Lactobacillus, Flavobacterium, Prevotela, Porphyromonas*, and *Gardnerella;* The mean proportion of proteobacteria was higher in leukocytospermic men	After intercourse, the seminal microbiome shifted the vaginal microbiome	High
Weng et al., 2014 (27) (China)	Cross-sectional	10 men with abnormal semen volume, 13 men with oligozoospermia, 12 men with asthenozoospermia, 44 men with teratozoospermia, 10 men with antisperm antibodies, And 18 men with; leukocytospermia	Semen	Sequencing V4 hypervariable region of 16S rRNA gene	Abnormal SA group: *Lactobacillus, Prevotella, Pseudomonas, Haemophilus, Finegoldia, Rhodanobacter, Corynebacterium* and *Streptococcus*		High
Sellami et al., 2014 (24) (Tunisia)	Cross-sectional	85 infertile men	Semen	Culture positive if concentration > 10⁴cfu/m, and PCR	Bacteriospermia in 7% of specimens; Culture: *Group B Streptococcus* (3.5%), *Enterococcus spp* (1.1%), *Staphylococcus aureus* (1.1%), and *Corynebacterium spp* (1.1%); PCR: *C. trachomatis* (15.2%), *N gonorrhea* (5.8%), *U. urealyticum* (5.8%), *M. genitalium* (5.8%), *U. parvum* (5.8%), and *M. hominis* (5.8%)	*C. trachomatis* associated with decreased sperm quality and increased apoptosis	High
Hou et al., 2013 (43) (China)	Cross-sectional	10 men with asthenozoospermia, 23 men with oligoasthenozoospermia, and 25 with oligozoospermia or azoospermia	Semen	Sequencing V1 and V2 hypervariable regions of 16S rRNA gene)	Overall: *Ralstonia, Lactobacillus, Corynebacterium, Streptococcus, Staphylococcus, Prevotella, Finegoldia*, and *Anaerococcus;* No differences among the groups	*Anaerococcus* had a negative association with sperm quality	Fair
Aghazarian et al., 2013 (50) (Iran)	Cross-sectional	171 men undergoing infertility evaluation	Semen	Not specified	Bacteriospermia in 36.2% of specimens; *Ureaplasma urealyticum + Gardnerella vaginalis* (25.8%), *Ureaplasma urealyticum* (19.4%), *G. vaginalis* (16.1%), *Enterococcus faecalis* (9.7%), *E. coli + E. faecalis* (1.6%)	No significant association between bacteriospermia and leukocytospermia; No significant differences in semen parameters in men with bacteriospermia	High
Domes et al., 2012 (51) (Canada)	Retrospective cohort	4935 samples from infertile men	Semen	Culture positive if concentration > 10³cfu/m	Bacteriospermia in 15% of specimens; *Staphylococcus aureus* (5%), *Enterococcus fecalis* (56%), *Escherichia coli* (16%), *Group B streptococcus* (13%), *Klebsiella pneumoniae* (2.2%), *Proteus mirabilis* (1.7%), *Citrobacter koseri* (1.5%), and *Morganella morganii* (1.3%)	Bacteriospermia associated with an increase in DFI; Elevated seminal leukocytes dominant factor associated with deterioration in semen parameters	High
Isaiah et al., 2011 (60) (Nigeria)	Cross-sectional	140 infertile men	Semen	Culture	Bacteriospermia in 65.7% of specimens; *Staphylococcus aureus* (28.3%), *Staphylococcus saprohyticus* (13%), *Pseudomonas aerouginosa* (6.5%), *Escherichia coli* (19.6%), *Proteus mirabilis* (10.8%), *Staphylococcus spp* (10.8%), *and Proteus vulgaris* (10.8%)	*Staphylococcus saprohyticus* and *Escherichia coli* associated with altered sperm motility and morphology; Significant (p<0.001) relationship between bacteriospermia, leukocytes, and total sperm count	High
Moretti et al., 2009 (61) (Italy)	Cross-sectional	236 men with bacteriospermia	Semen	Culture: positive if concentration > 10⁴cfu/ml if gram + and > 10⁵cfu/ml if gram	*E. faecalis* Bacteriospermia in 33.2% of specimens; (32.1%), *E.coli* (20.3%), *Streptococcus agalactiae* (13.4%), *U. urealyiticum* (11.8%), *Staphylococcus epidermidis* (9.7%), *Streptococcus anginosus* (9.3%), and *Morganella morganii* (3.2%)	Sperm concentration lower than in controls; progressive motility lower than controls except for samples positive for *S. agalactiae* and *S. anginosus*	High
Gdoura et al., 2008 (62) (Tunisia)	Cross-sectional	166 men undergoing infertility evaluation	Semen	Culture and PCR	Overall: *Chlamydia trachomatis* (41.4%), *Ureaplasma urealyticum* (15.5%), and *Mycoplasma hominis* (10.3%) Culture: *E. coli (*1.7%), *Streptococcus agalactiae* (0.9%), *Citrobacter diversus* (0.9%), *Enterococcus faecalis* (0.9%), and *Gardnerella vaginalis* (0.9%)	Bacteriospermia 56.9%; bacteria in 56% of semen samples by PCR; Bacteria in 5.2% semen samples by culture	High
Virecoulon et al., 2005 (45) (France)	Cross-sectional	534 male partners of infertile couples	Semen	Culture: positive if concentration > 10³cfu/ml	*Gardnerella vaginalis* (26.1%), *coagulase-negative staphylococci* (15.7%), *Streptococcus anginosus* (14.2%), *Ureaplasma urealyticum* (15.5%), *Enterobacteriaceae (E. coli, Proteus mirabilis), Corynebacterium spp, and Lactobacillus spp*	Sterile in 28.8%; polymicrobial flora in 49.3%; No relationship between the bacterial flora and leukocytospermia; Low titers of *U. urealyticum* in semen were not associated with a disturbance of the ecosystem	High
Levy et al., 1999 (24) (France)	Cross-sectional	92 male partners of infertile couples	Semen	Culture positive if concentration > 10⁴cfu/m, and PCR	Culture: *Ureaplama urealyticum* (13%) PCR: *Chlamydia trachomatis* (11%)	No relation between the presence of microorganisms in semen and serum antibodies	High
Debata et al., 1999 (63) (India)	Cross-sectional	197 infertile men	Semen	Culture	*Ureaplasma. urealyticum* (43%), *Mycoplasma hominis* (17%)	No association between *Ureaplasma* and sperm count; Bacteriospermia associated with altered sperm morphology	High
Kjaergaard et al., 1997 (44) (Denmark)	Cross-sectional	60 men with mild/moderate oligozoospermia and 26 men with severe oligozoospermia	Semen	Culture positive if concentration > 10³cfu/m, and PCR	Mild/moderate oligozoospermia: Commensals, *Ureaplasm. Urealyticum, Gardnerella vaginalis, Enterococcus faecalis*, Enterobacteriaceae, and *Mycoplasma;* Severe oligozoospermia: Commensals, *Ureaplasm. Urealyticum, Enterococcus faecalis, Gardnerella vaginalis*, Enterobacteriaceae, and *Mycoplasma*	No association between semen quality and microorganisms	High
Bussen et al., 1997 (49) (Italy)	Cross-sectional	88 male partners of infertile couples Group 1: 28 negative culture + 14 positive culture for microorganisms that colonize skin (considered control group); Group 2: 46 positive cultures	Semen	>100 colonies per plate	Bacteriospermia in 68% of specimens; *S. epidermidis* (33%): considered to be commensal *S. aureus* (9%); *E. coli* (8%); *Enterobacter* spp. (7%); *Group B streptococcus* (8%); *Corynebacteria* (8%)	No differences in sperm concentration, count, sperm morphology, and fertilization rates between groups	High
Shalika et al., 1996 (64) (USA)	Cross-seccional	342 male partners of infertile couples	Semen	Culture	Bacteriospermia in 32% of specimens; *Culture: S. aureus* (3%), *Enterococcus spp* (23%)*, Ureaplasma spp* (11%)*, E. coli (*3%), *Proteus mirabillis* (0.5%)*, and Streptococcus spp* (2%)	*Enterococcus spp* did not adversely affect IVF pregnancy rate; *E. coli, S aureus*, and *Ureaplasma urealyticum* potentially affecting IVF pregnancy rates	High
Eggert-Kruse et al., 1995 (48) (Germany)	Cross-sectional	126 male partners of infertile couples	Semen	Culture: positive if concentration > 10^6^cfu/ml	*Peptococcus sp* (38.1%), *Peptostreptococcus sp* (32.5%), *Veillonella spp* (27.8%), *Lactobacillus spp* (20.6%), *Bacterioides spp (*7.9%: *B. disiens, B.capillosus, B. ruminicola, B. bivius)*, *Propionibacterium spp* (7.1%), *Fusobacterium spp* (3.2%: *F. varium, F. mortiferum, F. nucleatum), Gardnerella vaginalis* (3.1%), and *Actinomyces spp (*1.6%: *A. meyeri, A. viscosus);* Gram-negative non-identified anaerobic rods (5.6%); Anaerobic bacteria not identified (11.9%) *Mycoplasma hominis* (6.1%); *Ureaplasma urealyticum* (21.2%)	99% of samples colonized with anaerobic; 71% potentially pathogenic species; Potentially pathogenic aerobic microorganisms more frequent in oligozoospermia group; *Bacteroides spp* and *Fusobacterium spp* more frequent in the asthenozoospermia and teratozoospermia groups (not statistically significant)	Fair

AT, asthenoteratozoospermia; OA, Obstructive Azoospermia; OAT, oligoasthenoteratozoospermia; H, hyperviscosity; iNOA, idiopathic non-obstructive azoospermia; SA, semen analysis; SDF, Sperm DNA Fragmentation; DFI, sperm DNA fragmentation index; PCR, polymerase chain reaction; cfu, colony forming units; HPV, human papillomavirus; SAM, S-adenosyl-L-methionine.

### Oligozoospermia

Oligozoospermia, characterized by a sperm concentration below the WHO reference limit (e.g., 16 x 10^6^ sperm/mL) (9, 65), was associated with specific bacterial genera in the study of Okwelogu and colleagues (34). *Prevotella*, *Escherichia*, *Lactobacillus*, *Shuttleworthia*, and *Serratia* were the most abundant genera in oligozoospermic men (34). This observation was corroborated by Lundy and colleagues, who described an inverse association between seminal abundance of *Prevotella* and sperm concentration (33).

### Asthenozoospermia

Bacterial presence in semen significantly affects motility (66), a critical component of a basic semen analysis assessment. Asthenozoospermia is typically defined as having less than 30% progressive spermatozoa or less than 42% total motility (9, 65, 67). Yang and colleagues showed that men with asthenozoospermia exhibited an increased abundance of *Sneathia, Ralstonia, Ureaplasma, Bacteroides*, and *Chryseobacterium* (35). Moreover, in men with oligoasthenozoospermia, the genera *Ralstonia, Oscillospira, Parabacteroides, Lachnospira*, and *Phascolarctobacterium* were more abundant. Notably, the authors reported an increased prevalence of *Lactobacillus* in men with astheno- or oligoasthenozoospermia compared to controls with normal basic semen analysis parameters, suggesting *Lactobacillus* as a potential bacterial biomarker for asthenozoospermia (receiver operating characteristics value of 0.841) (35). Similarly, Yao and colleagues found a *Lactobacillus*-enriched seminal microbial community prevailing in men with asthenozoospermia (31). In semen samples from men undergoing *in vitro* fertilization (IVF), Štšepetova and colleagues noted negative associations between sperm motility and the phyla *Bacteroidetes* and *Proteobacteria*, as well as the classes *Alphaproteobacteria* and *Sphingobacteria* (68). In contrast, another study found that the seminal abundance of *Pseudomonas*, a proteobacteria, was directly associated with total motile sperm count (33).

### Oligoasthenoteratozoospermia

Oligoasthenoteratozoospermia (OAT), characterized by abnormalities in the three primary semen analysis parameters (i.e., sperm concentration, motility, and morphology) (9, 67), is indicative of severe impairment of spermatogenesis and is linked to reduced chances of natural pregnancy (69). Monteiro and colleagues associated OAT with the presence of Cyanobacteria and Fusobacteria, an increased prevalence of *Pseudomonas, Klebsiella, Aerococcus, Actinobaculum*, and *Neisseria*, as well as a decreased prevalence of *Lactobacillus* and *Propionibacterium* (40).

### Azoospermia

Azoospermia is the lack of spermatozoa in the ejaculate (70). Examining men undergoing IVF, Okwelogu and colleagues found *Lactobacillus*, *Enterococcus*, *Corynebacterium*, *Veillonella*, and *Gardnerella* were the most abundant genera in azoospermic men (34). However, the authors did not specify the cause of azoospermia.

### Semen quality in general

Several studies investigated the microbiome in men with abnormalities in any basic semen analysis parameters, often referred to as low-quality semen. Hou et al. found no significant differences in the seminal bacterial composition between healthy semen donors and infertile men with abnormal basic semen analysis parameters (43). However, they did observe a negative association between semen quality and the presence of Anaerococcus. In contrast, Weng et al. demonstrated that a Prevotella-predominant bacterial community was associated with low-quality semen (27). Similarly, Baud et al. found that the *Prevotella* genus was significantly enriched in the semen of men with abnormal semen analysis parameters (36).

Furthermore, Lundy et al. reported an increased prevalence of *Aerococcus* and decreased Collinsella in semen samples from infertile men compared to fertile controls (33). In this study, male infertility was defined as the presence of altered basic semen parameters and an inability to father a child after 12 months of trying. Along these lines, Bukharin et al., also studying infertile men, demonstrated that *Staphylococcus*, *Corynebacterium*, *Enterococcus*, *Streptococcus*, and *Escherichia* were the most prevalent genera in the semen (32).

### Leukocytospermia

Leukocytospermia, characterized by the presence of >1.0 million leukocytes per mL of semen (71), is classically associated with genitourinary tract infection since bacteriospermia can trigger the recruitment of seminal leukocytes (10). However, other conditions, such as exposure to vaginal products during intercourse, smoking, genitourinary procedures, and autoimmunity, may increase the number of leukocytes in semen (51). In most studies employing standard culture techniques, the presence of bacteria in the semen of asymptomatic men was not associated with an increase of seminal leukocytes (45, 50–52), even when leukocytospermia was defined with low cutoff values (e.g., 0.2 x 10^6^ leukocytes/mL) (62). Despite that, some authors have reported associations between leukocytospermia and bacteriospermia (38, 44, 60, 61). For instance, Yao et al., using NGS to assess the seminal microbiome, reported that a *Streptococcus*-enriched bacterial community predominated in men with leukocytospermia (31). The authors also found an increased prevalence of Bacteroidetes associated with leukocytospermia. Additionally, Štšepetova et al. showed that in men undergoing IVF, *Staphylococcus* sp. was associated with leukocytospermia (68). However, Lundy and colleagues found an inverse association between *Aerococcus* abundance and leukocytospermia. Nevertheless, when comparing the seminal microbiome between infertile men with and without leukocytospermia, no differences were observed in measures of bacterial diversity (33).

### Oxidative stress and sperm DNA fragmentation

Oxidative stress and sperm DNA fragmentation are commonly observed in infertile men and may result from the activation of seminal leukocytes (19, 72–75). When using NGS to evaluate the seminal microbiome of men with elevated oxidative stress (oxidation-reduction potential >1.34 mV/10^6^ sperm/ml), Lundy et al. reported modest differences in three taxa (*Serratia, Streptococcus and Curvibacter*) (33). Conversely, using culture-based methods, Zeyad and colleagues did not find differences in sperm DNA fragmentation levels between men with or without bacteriospermia (38). However, in a large study including nearly 5,000 infertile men, Domes et al. identified a negative association between culture-positive semen and sperm DNA integrity (41). In line with this, when assessing only healthy men with normal basic semen analysis parameters, Fraczek and colleagues reported increased sperm DNA damage in those with positive semen culture but no increase in oxidative stress markers (42).

### Inflammatory markers

Bacteria in the genitourinary tract may lead to inflammatory responses mediated by various cytokines produced by leukocytes (72). Hence, it is reasonable to assume that the seminal microbiome can influence the production of these inflammatory mediators. Bukharin et al. demonstrated that *Staphylococcus* isolated from the semen of healthy men degraded IL-10 and IL-17 more intensely than those from the semen of infertile men (32). Additionally, *Enterococcus* from infertile men reduced IL-1 levels, and *Corynebacterium* from these individuals reduced TNF-α levels to a greater extent than those isolated from healthy subjects (32). These findings suggest that the seminal microbiome can influence the host’s inflammatory response, at least locally. By contrast, culture-based studies did not establish an association between the presence of bacteria in semen and seminal levels of inflammatory markers (50, 76).

## Epididymal and testicular microflora

Evaluating the epididymal or testicular microbiome requires harvesting biofluids or tissue samples from these organs. In this context, Alfano et al. conducted a study using testicular samples from men with idiopathic non-obstructive azoospermia (iNOA) and normozoospermic men who underwent orchiectomy (37). Employing NGS to sequence the V3 to V5 hypervariable regions of the 16S rRNA gene with NGS, the authors made a groundbreaking discovery, revealing that the testicular compartment is not sterile. In the testicular tissue of men with normal spermatogenesis, they identified the phyla *Actinobacteria, Bacteroidetes, Firmicutes, and Proteobacteria*. However, despite the increased presence of bacterial DNA in testicular samples from men with iNOA, only the phyla *Actinobacteria* and *Firmicutes* were identified in these samples.

Furthermore, the testicular microbiome of NOA men with complete germline cell aplasia exhibited reduced bacterial richness and diversity, with a dominance of the *Actinobacteria* phylum and the absence of *Clostridia*. Similarly, Molina et al. utilized testicular samples from sperm retrieval procedures in men with azoospermia, severe oligoasthenozoospermia, or high DNA fragmentation to study the testicular microbiome (53). The authors also observed low levels of bacteria and identified ten genera specific to the testicles, including *Blautia (phylum Firmicutes), Cellulosibacter (Firmicutes), Clostridium XIVa (Firmicutes), Clostridium XIVb (Firmicutes), Clostridium XVIII (Firmicutes), Collinsella (Actinobacteria), Prevotella (Bacteroidetes), Prolixibacter (Bacteroidetes), Robinsoniella (Firmicutes), and Wandonia (Bacteroidetes).* Notably, despite stringent antiseptic measures, contamination accounted for 50–70% of all detected bacterial reads, suggesting that sperm retrieval from the testes is not performed under sterile conditions (53).

Using a different strategy, Lundy et al. demonstrated that *Collinsella* and *Staphylococcus* were prevalent in semen samples from healthy fertile men and depleted in samples from men who underwent vasectomy, implying that these two genera are constituents of the testicular or epididymal microbiome (33). To date, no studies have examined the epididymal microbiome.

## Other factors that can affect the seminal microbiome

### Diet (gut microbiome)

High-fat (HFD) and high-sugar “Western” diets have been associated with obesity, metabolic disorders, and alterations in gut microbiota composition in both humans and animals (3). However, the impact of HFD-induced dysbiosis on reproductive function remains unclear. In a study by Zhang et al., significant differences in the bacterial composition of the gut microbiota were observed between the HFD and normal diet groups (77). The HFD was associated with a decreased abundance of Bacteroidetes and Verrucomicrobia and an increased abundance of Firmicutes and Proteobacteria (3). Notably, the HFD resulted in reduced sperm concentration and motility, along with a decrease in spermatocyte and round spermatid numbers. Analysis of the gut microbiota in the HFD group revealed an increased abundance of *Bacteroides*, *Prevotella*, *Rikenella*, and *Lactobacillus*. The authors also analyzed fecal samples from healthy semen donors and infertile men with asthenozoospermia, oligozoospermia, and teratozoospermia, demonstrating a similar strong negative correlation between sperm motility and the combined abundance of Bacteroides and Prevotella. Moreover, *Prevotella copri*, a dominant species within Prevotella, was implicated in spermatogenic defects. These findings suggest a potential role of HFD-induced gut microbiota dysbiosis in impairing spermatogenesis and sperm motility.

### Sexual habits

A culture-based study demonstrated that men who never had sexual intercourse exhibited lower total seminal bacterial concentration and diversity than sexually active men (78). Nelson et al. applied sequencing of the V1-V9 sub-regions of 16 S rRNA alleles to evaluate the coronal sulcus microbiome from eighteen healthy 14–17 year-old teens (79). The authors reported that some taxa associated with bacterial vaginitis including *Mycoplasma*, *Ureaplasma*, and *Sneathia* were detected only in participants with sexual experience, mainly vaginal intercourse and fellatio. Moreover, studying men who have sex with men, Liu et al. observed that bacteria in the semen of these men overlapped with those previously described in the vagina, including *Streptococcus*, *Corynebacterium*, *Staphylococcus*, *Prevotella* and *Mycoplasma* (80). These finding suggest that partnered sexual activity influences on the composition of the seminal microbiome. Unfortunately, there is no data in the current literature regarding the association of specific modalities of sexual activity to changes in the seminal microbiome.

### Sexually transmitted infections

Human papillomavirus (HPV) infection has been associated with reduced semen quality (17, 79, 81). The mechanisms underlying this association are unclear but may include apoptosis of sperm cells, sperm DNA damage, and the production of antisperm antibodies. Additionally, HPV-positive semen samples exhibited higher *Moraxellaceae*, *Streptococcus*, and *Peptostreptococcus* abundances than HPV-negative semen samples (80). Notwithstanding these observations, the authors of the above study did not perform semen analysis to assess the impact of these alterations on semen quality.

Furthermore, Human Immunodeficiency Virus (HIV) has been shown to induce changes in the seminal microbiome. Liu et al. demonstrated that men with HIV infection had decreased semen microbiome diversity and richness, which were restored after six months of antiretroviral therapy (82). The semen bacterial load was associated with pro-inflammatory semen cytokines and semen viral load, suggesting a role of the semen microbiome in HIV sexual transmission (82).

## Impact of seminal microbiome on the female genital tract

The seminal microbiome influences the microflora of the female genital tract. A well-established example of such influence is the association between bacterial vaginosis, a dysbiotic condition, and frequent vaginal intercourse (83). A study examining risk factors for bacterial vaginosis in women with and without HIV infection demonstrated that the presence of spermatozoa in Gram-stained vaginal smear samples, which serves as a biological marker of recent exposure to semen, was the only common factor in both groups (84).

Furthermore, the production of H_2_O_2_ by certain vaginal lactobacilli is essential for maintaining a healthy vaginal environment (85). Having more than two sexual partners during the past year has been identified as a risk factor for the absence of H2O2-producing lactobacilli among women with bacterial vaginosis (86). Several studies that simultaneously assessed the seminal and vaginal microbiomes of sexual partners have confirmed this finding. For instance, Okwelogu et al. found that couples shared 56% of predominant genera, suggesting that the composition of the reproductive tract microbiota, whether healthy or dysbiotic, could influence the microbial composition of their sexual partners (34). Similarly, Campisciano et al. demonstrated that couples with infertility of known causes exhibited an overlap in the bacterial composition of their seminal and vaginal microbiomes, including an increased prevalence of *Staphylococcus* and *Streptococcus* genera (54). The authors also noted a higher abundance of *Lactobacillus gasseri* in the semen of couples with unexplained infertility than those with explained infertility. Using PCR and culture-based techniques, Borovkova et al. found that up to seven new species could be introduced, and the same number removed from vaginal microflora after intercourse (87). Additionally, using culture-based methods, Ricci et al. found that 61% of couples with positive cultures in both partners shared at least one genital pathogen (52).

## Impact of seminal microbiome on the reproductive outcomes

### Natural pregnancy

Early studies utilizing culture-based methods failed to identify differences in the microbial patterns in the ejaculate of men from couples who achieved natural pregnancy compared to those who did not (49, 64, 76). In a study by Eggert-Kruse et al., anaerobic and “potentially pathogenic” bacteria were cultured in 94.7% and 84.2% of the fertile men, respectively. Furthermore, there was no association between microbial colonization and natural pregnancy after a 6-month follow-up (76).

### Assisted reproduction technology

Semen and vaginal cultures are typically carried out before assisted reproduction technology (ART). However, interpreting positive cultures in asymptomatic patients can be challenging due to the possibility of contamination. Nonetheless, specific pathogens, such as *E. faecalis*, *U. urealyticum*, *M. hominis, G. vaginalis, and E. coli*, were more prevalent in the genital tract of couples that had experienced IVF failure (52). Additionally, Zeyad et al. reported a weak negative correlation between bacteriospermia and fertilization rates in couples undergoing IVF (r=−0.239, p<0.05) (38). Interestingly, sperm preparation techniques like swim-up and density gradient (88), commonly used to process semen for ART, can reduce bacteria counts in asymptomatic infertile men, but total clearance is rarely achieved (89). Thus, it seems evident that ART is commonly performed in a non-sterile environment despite taking precautions to prevent sample and equipment contamination (90).

**Figure 3:**
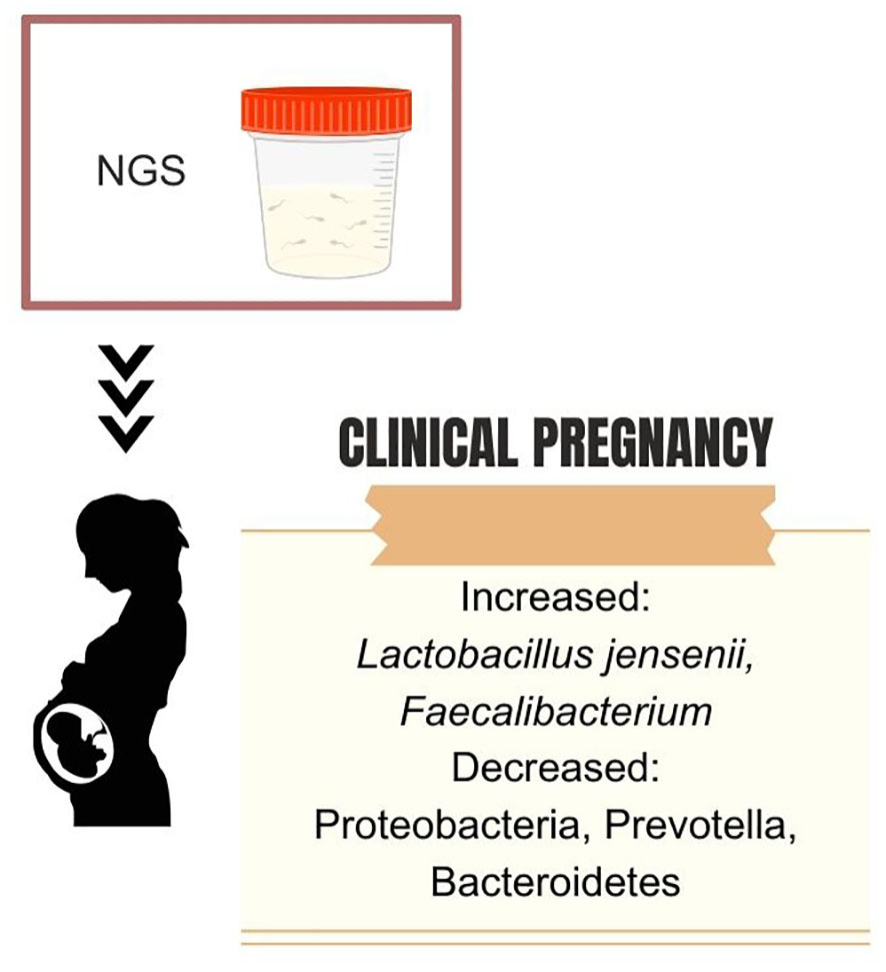
Dominant seminal microbiota genera associated with pregnancy success after assisted reproductive technology.

**Figure 4:**
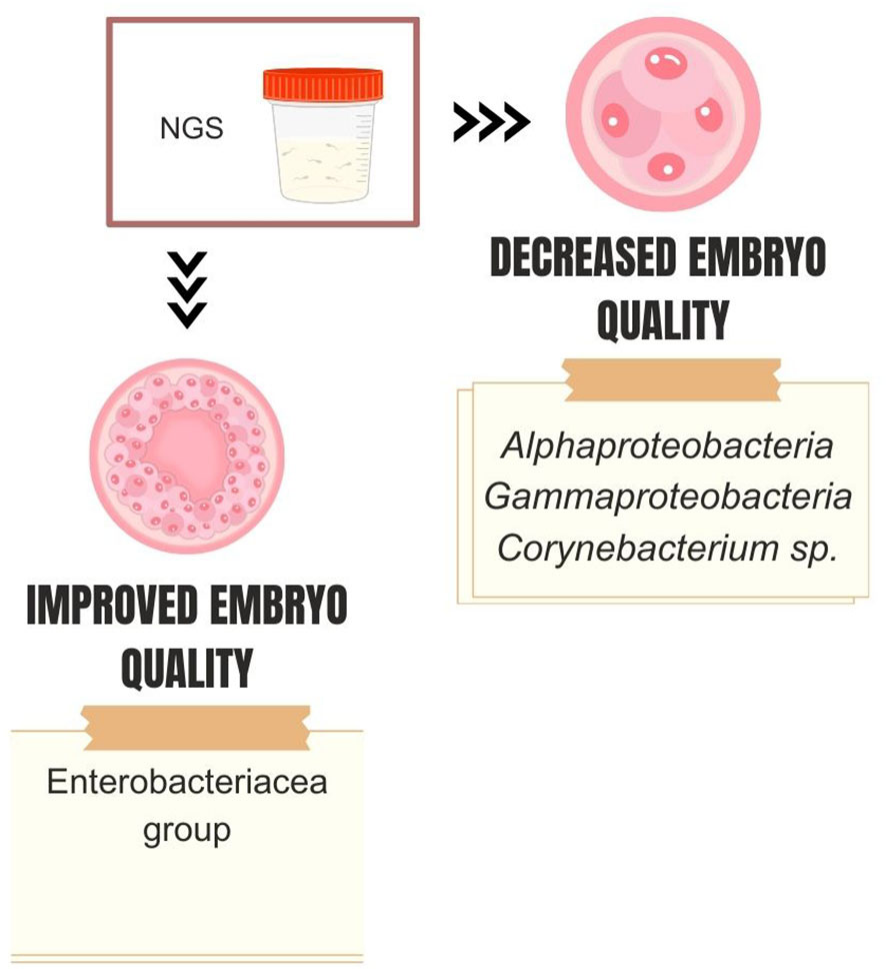
Dominant seminal microbiota genera associated with embryo quality.

NGS studies corroborate this idea and have reported associations between specific types of seminal microbiome and *in vitro* fertilization/intracytoplasmic sperm injection (IVF/ICSI) outcomes (**Figure 3**). Štšepetova et al. investigated the microbiome of raw semen, processed semen, incubated sperm, and IVF culture media from 50 couples undergoing IVF (68). The authors utilized sequencing of the V2 and V3 hypervariable region of the 16S rRNA gene and real-time PCR. They observed decreasing bacterial reads count as semen samples underwent processing (i.e., raw > washed >incubated). The most abundant genera of bacteria in raw semen were *Lactobacillus*, *Incertae sedis XI*, *Staphylococcus*, and *Prevotella*. Processed semen samples exhibited a more heterogeneous microbial composition. Higher counts of *Alphaproteobacteria* and *Gammaproteobacteria* in washed sperm, as well as *Corynebacterium* sp. in raw semen samples, were associated with reduced embryo quality. Conversely, couples with increased embryo quality had a higher mean proportion of the *Enterobacteriaceae* group in raw semen (**Figure 4**). Bacterial reads were detected in IVF culture media in 8% of the samples via NGS and more than 70% by the real-time PCR method, with *Lactobacillus* and *Phyllocterium* being the most frequent genera.

**Figure 5:**
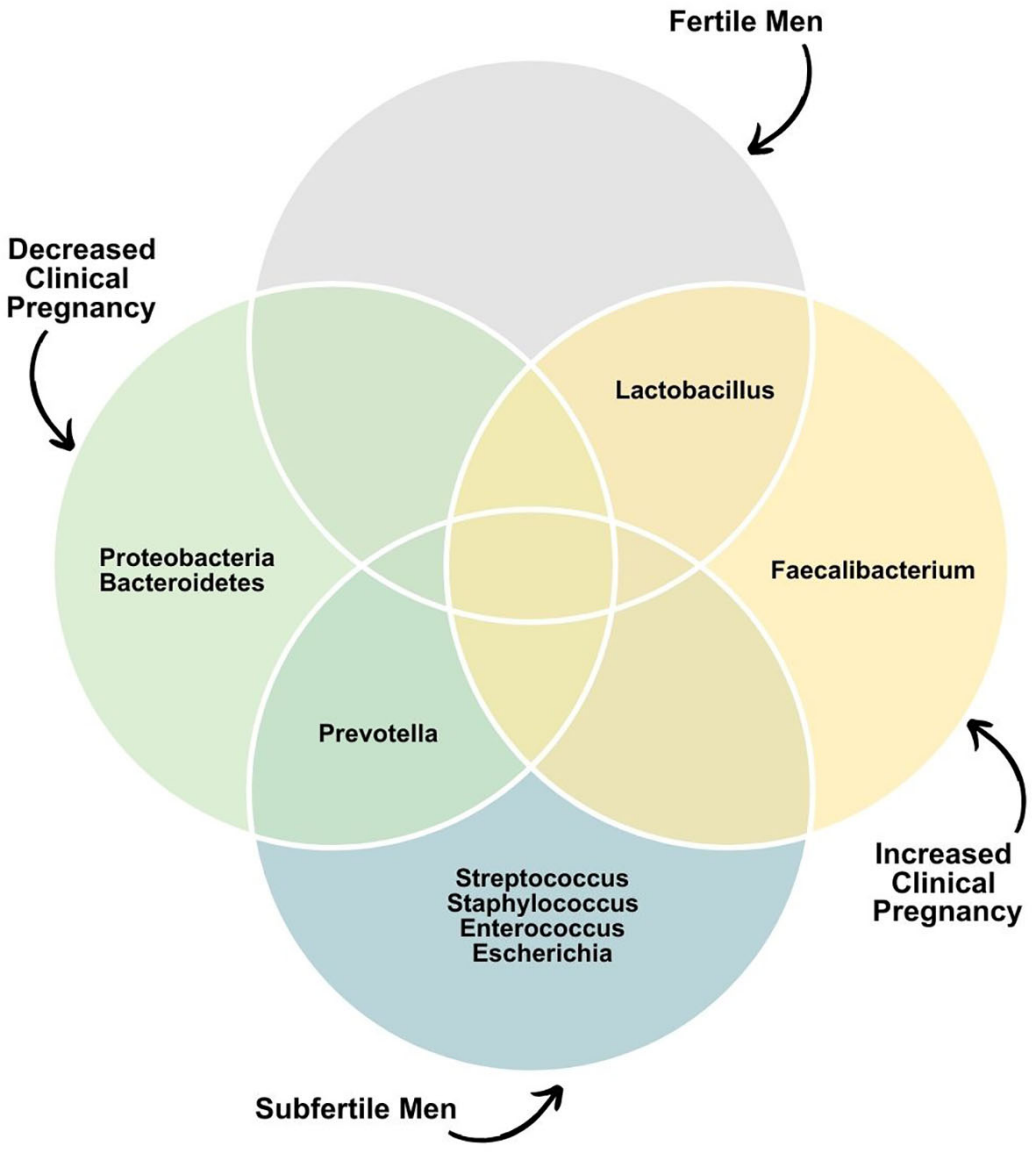
Dominant seminal microbiota phyla or genera associated with fertility status and outcomes.

Nevertheless, bacteria in IVF culture media do not seem to influence pregnancy rates. Utilizing sequencing of the V4 region of the 16S rRNA, Okwelogu et al. demonstrated that semen samples from couples with clinical pregnancy after ICSI exhibited increased colonization by *Lactobacillus jensenii* group and *Faecalibacterium*, along with a decreased prevalence of *Proteobacteria*, *Prevotella*, *Bacteroidetes* taxa compared to those with adverse outcomes (34). Conversely, a study applying sequencing of variable regions 3 and 4 of the 16S rRNA found no differences in seminal microbiome composition and diversity between male partners of couples that had or did not have a successful pregnancy after intrauterine insemination (91). On the female side, a recent analysis of the endometrial microbiome of women undergoing IVF demonstrated that 73.9% of the endometrial samples assessed with NGS were colonialized by one or more microbes, further highlighting the fact that human reproduction often happens in the presence of a bacterial microbiota (92). **Figure 5** summarizes the main bacterial phyla or genera associated with fertility status and outcomes.

## Future directions and recommendations for semen microbiome studies

The emerging field of seminal microbiota research has illuminated the intricate microbial communities residing in the male reproductive tract. While significant progress has been made in characterizing these microorganisms and their potential functions of these microorganisms, several critical areas detailed below merit further exploration (93).

1. **Standardized Protocols**: To ensure the reliability and reproducibility of results, it is imperative to establish standardized protocols for sample collection, handling, DNA extraction, and NGS analysis. Future research should formulate guidelines and best practices to mitigate technical variations and biases that may arise during these processes. This will facilitate robust comparisons between studies and enable the integration and comparison of findings across different research groups.
2. **Optimal Variable Regions:** The choice of variable regions within the 16S rRNA gene for sequencing can impact the accuracy and resolution of results. Future studies should aim to pinpoint the most informative variable regions specific to the seminal microbiota. This will help establish a standardized sequencing approach, enhancing comparability across studies and facilitating meta-analyses.
3. **Shotgun Metagenomics**: While most research has focused on bacterial communities, the seminal microbiota likely includes viruses and fungi. Future investigations should leverage shotgun metagenomics approaches to comprehensively identify and characterize these components. This will provide a complete understanding of the microbial landscape and its potential role in male infertility and semen abnormalities.
4. **Contamination Mitigation:** Contamination poses a significant challenge in microbiota research. Future studies should prioritize stringent measures to prevent and detect contamination at each experimental stage. This includes incorporating negative controls during sample collection, DNA extraction, library preparation, and sequencing. Stringent quality control measures will enhance the reliability of results and minimize the impact of potential contamination on data interpretation.
5. **Pathogenic strain determination**: It is known that some bacteria species can have pathogenic and non-pathogenic strains. Thus, it is of utmost importance to differentiate the strains that have the potential to cause harm from those that are commensals. This subtyping can be done using NGS (64), and coupled with data from databases such as the National Center for Biotechnology Information Pathogen Detection, this approach can better identify “friends and foes”.
6. **Functional role of seminal microbiota**. Few studies have delved into the role played by the male genital tract microbiome and who it interacts with spermatogenesis, but it is plausible that this microbiota regulates the immune microenvironment of testis, playing a role in providing nutrients, regulating the testicular immune microenvironment, and modulating signal transduction (88, 89). To advance in this field, further studies should focus not only on describing the components of the seminal microbiome, but also to assess their function in this system by using *in vitro* and ex vivo experimental systems for studying host–microbiome interactions, similar to what has been used to study gut and respiratory microbiomes (90).
7. **Multi-site Investigation**: Given that the seminal microbiome likely originates from multiple sites within the reproductive tract, simultaneous assessment of the microbiome composition of each of these organs (i.e., testis, epididymis, vas deferens, prostate, seminal vesicles, urethra, and penis) may provide deeper insights into their relevance to male infertility conditions, enabling more tailored treatment.
8. **Longitudinal Studies**: Longitudinal research is crucial for capturing dynamic changes in the seminal microbiota over time and understanding its potential impact on male fertility. Future research should prioritize longitudinal study designs to explore temporal variations in the microbiota composition and function within individuals and across different stages of reproductive health. This will elucidate the role of seminal microbiota in physiological and pathological conditions and its contribution to infertility and semen alterations.
9. **Prospective Studies**: Prospective studies are necessary to establish a direct link between seminal microbiota and reproductive outcomes. These studies should involve monitoring the male reproductive tract microbiota in men attempting natural conception or undergoing ART. Researchers might unravel the seminal microbiome’s potential impact on fertility and ART success by correlating microbiota profiles with pregnancy rates, embryo development, and other ART outcomes.
10. **Impact of Male Infertility Causes**: Male infertility can stem from various causes, including genetic factors, hormonal imbalances, infections, and structural abnormalities. Future research should investigate the specific influence of different infertility causes on the seminal microbiome. This will shed light on whether distinct microbial signatures are linked to specific infertility etiologies and guide the development of targeted therapeutic strategies tailored to individual patients.
11. **Effects of Commonly Prescribed Drugs**: Several drugs and treatments are commonly prescribed for male infertility management, such as vitamin supplements, antibiotics, and hormonal therapy. Future research should explore whether and how these interventions impact the seminal microbiome. Understanding the effects of these therapeutic agents on microbial communities will provide insights into their potential contributions to fertility outcomes and guide the development of more personalized treatment regimens.

## Conclusions

The exploration of the seminal microbiome has opened a fascinating realm of research, shedding light on the intricate microbial communities residing within the male reproductive tract. This emerging field has revealed a complex interplay between these microorganisms and male fertility, semen quality, and their potential influence on female reproductive health. The evidence compiled from various studies using culture-based and NGS techniques has provided valuable insights into these microbial communities’ composition, dynamics, and potential functions. One of the key takeaways from this review is the pressing need for standardized protocols and best practices in sample collection, DNA extraction, and NGS analysis. By establishing rigorous methodologies, the scientific community can ensure the reliability and reproducibility of results, fostering more robust comparisons between studies and facilitating meta-analyses. Additionally, the choice of variable regions within the 16S rRNA gene for sequencing and the application of shotgun metagenomics for a comprehensive assessment of viruses and fungi within the seminal microbiota are vital considerations for future research. Prospective and longitudinal studies and investigations into the impacts of various male infertility causes and commonly prescribed drugs hold promise for unraveling the intricate relationships between the seminal microbiome and male reproductive outcomes. This knowledge enhances our understanding of male fertility and paves the way for personalized interventions and treatments tailored to individual patients. Exploring the seminal microbiome represents a dynamic and rapidly evolving field poised to advance our comprehension of male reproductive health and potentially revolutionize clinical approaches to male infertility and semen alterations.

## Author contributions

FL: Writing – original draft. MV: Writing – original draft. FC: Funding acquisition, Writing – review & editing. AC: Funding acquisition, Writing – review & editing. CA: Funding acquisition, Writing – review & editing. SE: Conceptualization, Methodology, Project administration, Supervision, Writing – review & editing.
